# Effect of Health Information Technologies on Glycemic Control Among Patients with Type 2 Diabetes

**DOI:** 10.1007/s11892-018-1105-2

**Published:** 2018-10-18

**Authors:** Yilin Yoshida, Suzanne A. Boren, Jesus Soares, Mihail Popescu, Stephen D. Nielson, Eduardo J. Simoes

**Affiliations:** 1 Department of Health Management and Informatics, School of Medicine, University of Missouri-Columbia, CE707 CS&E Bldg., One Hospital Drive, Columbia, MO USA; 2 Missouri Cancer Registry and Research Center, University of Missouri-Columbia, Columbia, MO USA; 3 Centers for Disease Control and Prevention, Division of High-Consequences Pathogens and Pathology, Prion and Public Health Office, Atlanta, GA USA; 4 Mercy Medical Center, Sioux City, IA USA

**Keywords:** Health information technologies, Glycemic control, Glycated hemoglobin level, Type 2 diabetes

## Abstract

**Purpose of Review:**

This study was to present meta-analysis findings across selected clinical trials for the effect of health information technologies (HITs) on glycemic control among patients with type 2 diabetes.

**Recent Findings:**

HITs may be promising in diabetes management. However, findings on effect size of glycated hemoglobin level (HbA1c) yielded from HITs varied across previous studies. This is likely due to heterogeneity in sample size, adherence to standard quantitative method, and/or searching criteria (e.g., type of HITs, type of diabetes, specification of patient population, randomized vs. nonrandomized trials).

**Summary:**

We systematically searched Medline, Cumulative Index of Nursing and Allied Health Literature (CINAHL), and the Cochrane Library for peer-reviewed randomized control trials that studied the effect of HITs on HbA1c reduction. We also used Google Scholar and a hand search to identify additional studies. Thirty-four studies (40 estimates) met the criteria and were included in the analysis. Overall, introduction of HITs to standard diabetes treatment resulted in a statistically and clinically reduced HbA1c. The bias adjusted HbA1c reduction due to the combined HIT interventions was − 0.56 [Hedges’ g = − 0.56 (− 0.70, − 0.43)]. The reduction was significant across each of the four types of HIT intervention under review, with mobile phone-based approaches generating the largest effects [Hedges’ g was − 0.67 (− 0.90, − 0.45)]. HITs can be an effective tool for glycemic control among patients with type 2 diabetes. Future studies should examine long-term effects of HITs and explore factors that influence their effectiveness.

**Electronic supplementary material:**

The online version of this article (10.1007/s11892-018-1105-2) contains supplementary material, which is available to authorized users.

## Introduction

Diabetes is now a world epidemic. It affected 8.8% (415 million) adult population (age 20–79 years) in 2015 worldwide and the prevalence will rise to 10.4% (642 million) by 2040 (10.4%) [1]. Diabetes is also the seventh leading cause of deaths in the world. Around 1.6 million people died due to diabetes in 2016 [2]. Successful glycemic control helps to prevent and reduce complications of diabetes, including cardiovascular disease, kidney disease, blindness, neuropathy and limb amputation, and reduce death related to the disease [3, 4]. However, to maintain optimal glycemic control requires ongoing monitoring and treatment, which can be costly and challenging. Research has demonstrated the association between self-monitoring and education and improved diabetes outcomes [5]. Importantly, patients with diabetes are encouraged to manage their health as best as they can to minimize complications [5].

To improve diabetes management, development of innovative self-care strategies is warranted. Advances in health information technologies (HITs) have introduced approaches that support effective and affordable health care delivery and patient education. Technologies in mobile, computer, e-mail, and internet approaches have shown evidence in enhancing chronic disease management [6, 7], suggesting great potential for diabetes management technologies. One successful example of HIT-enabled self-care is the home asthma telemonitoring system, which provides patients with continuous individualized help in the daily routine of asthma self-care [8]. Web-based applications also have shown potential to increase disease knowledge and social support to different patient groups (e.g., breast cancer patients, HIV patients) by providing a computer interactive system containing information, social support, and problem solving [9]. Additionally, the use of mobile technology devices such as personal digital assistants (PDAs) and cellular phones in health care is increasing as they have advantages of bringing additional resources to the care and changing the location of care. Mobile health promotion or wellness applications have primarily addressed smoking cessation [10], nutritional intake [11], and vaccinations [12]. These applications have also been tested in care of asthma [13] and chronic obstructive pulmonary disease [14]. Mobile phone short message service (SMS) has been used to remind patients of scheduled visits, deliver test results, and monitor side-effects of treatment [15–17]. Many of these aforementioned HITs have also been applied or attempted to be utilized in diabetes self-care, however, a comprehensive examination of the effectiveness, specifically across multiple types of HITs on glycemic control has not been clearly documented [18].

The benefits of HITs in diabetes management seems well illustrated in theory—they have the potential to empower patients and support a transition from a role in which the patient is the passive recipient of care services to an active role in which the patient is informed, has choices, and is involved in the decision-making process [19••]. They are also designed to promote communication and relationships between clinicians and patients, and overcome geographical barriers and logistical inconvenience when seeking health care services [20]. However, in reality, adopting these technologies still involves challenges and the rate of use is limited [20, 21•], since their effectiveness relative to usual care is yet to be determined [21•]. A growing number of randomized controlled trials (RCTs) have been conducted aiming to assess the effectiveness of HIT methods or tools in diabetes management. However, results have been mixed [22–24]. Previous reviews in this field suggested that HITs have the potential to improve glycemic control and other outcomes [21•, 25–28]. However, effects size specific to glycated hemoglobin (HbA1c) varied between studies, mean difference ranging from − 0.28 to − 0.44% [21•, 25–28]. Many of these studies are limited and restricted by modest sample size [27], lack of adherence to standard quantitative methods [23], inadequate attention to heterogeneity across studies [29], or lumped nonrandomized and randomized trials together into evaluation [22, 23, 25, 30, 31]. Some did not distinguish results for type 1 or type 2 diabetes [21•, 22, 23, 31–33]. Some exclusively examined one single type of HITs [21•, 33–36] or specified study’s searching criteria to a particular patient population [27, 37]. The objective of the present study was to systematically review and examine the current state of evidence concerning the effects of HITs on patient outcomes, particularly on glycemic control among patients with type 2 diabetes. We included a wide array of HITs and focused only on type 2 diabetes. Type 2 diabetes accounts for more than 90% of all diabetes cases [38]. Compared to patients with type 1 who often rely more on insulin administration, those with type 2 diabetes, especially in early stage of the disease, may be more sensitive to lifestyle modifications reinforced by HITs [39]. We also exclusively focused on reviewing rigorously designed randomized control trials, which is believed to involve fewer biases compared to nonrandomized trials.

## Methods

### Information Sources and Search Strategy

We systematically searched Medline for eligible articles from 1946 to December 2017, using combinations of the following MeSH (M) and textword (TW) search terms: (1) Diabetes Mellitus Type 1 (M), Diabetes Mellitus Type 2 (M), diabetes (TW), diabetes mellitus (M), prediabetic state (M), prediabetes (TW), (2) telemedicine (M), mHealth (TW), cell phone (M), cell phone$ (TW), mobile phone$ (TW), telehealth (TW), eHealth (TW), internet (M), ambulatory monitoring (M), and wearable$ (TW). Similar searches were conducted in Cumulative Index of Nursing and Allied Health Literature (CINAHL), and the Cochrane Library. We also used Google Scholar to identify additional studies not listed in the abovementioned databases. We also performed supplementary searches using references lists of eligible articles and relevant systematic review and other review articles we encountered.

### Eligibility Criteria

Articles were deemed eligible if they were peer-reviewed RCTs containing methodology and results sections that studied the effect of HITs including mobile phone-based, web-based, short message/text and other technologies on HbA1c among patients with T2D. Articles were excluded if they only included patients with type 1 diabetes, involved continuous glucose monitors, were feasibility trials, or were not written in English.

### Data Screening

A multistage screening process was used. Search results were first pooled and duplicates were removed. Next, article abstracts were screened for apparent relevance. Then the article texts were reviewed to confirm eligibility status. Articles extracted from reference lists underwent an identical process.

### Data Extraction

Following the screening process, data from eligible articles were extracted independently by two researchers. A coding manual was used to maintain reliable practices. The coding manual specified study characteristics (percentage of type 1 and type 2 patients, basic demographic data, geographic setting), intervention characteristics (mobile technology utilized, education provided in the intervention, intervention delivery personnel, equipment provided, intervention length), and clinical outcome (HbA1c). For the clinical outcome, additional data were extracted concerning the intervention’s treatment effect compatible with meta-analysis. Discrepancies were unanimously resolved before final data entry.

### Methodological Quality Assessment

The Cochrane Collaboration Risk of Bias Assessment tool was used to appraise the quality of each article by two reviewers. Six domains of bias (i.e., selection, performance, detection, attrition, reporting, and other) are included in the tool and risk scored as low, high, or unclear [40]. For each study, domain scores were summed to determine an overall score with risk of bias gauged low, unclear, or high [40]. Any discrepancies between assessors were discussed until consensus were reached.

We appraised the risk of selective reporting or publication bias by visual inspection of funnel plot symmetry of the standard error of each trial plotted against its estimated effect. This assumes that trials with larger sample sizes were less subject to publication bias. We further assessed the publication bias using a fail-safe *N* test [40].

### Quantitative Synthesis

RCTs containing methodology and results sections that studied the effect of mobile or potentially mobile HIT on type 2 diabetes were eligible for meta-analysis inclusion. We calculated a raw effect size measure of HIT on HbA1c (%) across 40 point estimates in 34 studies (i.e., mean difference in the HbA1c (%) changes from baseline to follow up time between intervention and control groups). We used Comprehensive Meta-analysis Version 3 statistical software to calculate two similar effect size measures modified in two ways [41, 42]. First, we calculated a standardized difference in means. Lastly, we calculated an effect size measure adjusted to bias attributed to the use of different populations across the 34 studies using a random-effect model (Hedges’ g effect size) [43]. For missing standard deviation (SD) values, we used an average of SDs from other studies in the same meta-analysis [44]. Heterogeneity of each model was assessed using Cochran’s Q and *I*^2^ statistics [43]. We considered heterogeneity to be greater than expected by chance alone if either the Cochran’s Q showed *P* < 0.05 or the *I*^2^ statistics was ≥ 50% [45]. In addition to the overall analysis of all included studies, we also examined stratified results according to the primary HIT tools used. These include mobile phone-based applications, web-based applications, SMS/Text and Others. “Others” is composed of telephone calls, devices such as video-phone, interactive voice system, PDA, wireless connected sensors, weight scale, step counter, and glucometer. We also present blood glucose reductions as estimated average glucose, or eAG, an American Diabetes Association recommended new term in diabetes management, eAG is calculated from the result of an HbA1c test [46]. It shows what an individual’s average blood sugar has been over the previous 2 to 3 months. Instead of a percentage, the eAG is the same units (mg/dl or mmol/L) as one’s blood glucose meter [46]. Reporting glucose control as eAG will assist health care providers and their patients in being able to better interpret the HbA1c value in units similar to what patients see regularly through their self-monitoring [46].

Although the effects of HIT on HbA1c is a mix of both HITs and standard diabetes care, including medication adherence and lifestyle modifications, in 16 of the reviewed trials, this effect was not clearly distinguished (i.e., treatment information in control group was not specified or standard care components were unclear in both intervention and control groups). For this reason, we repeated the overall synthesis analysis using data from the remainder 18 studies (53%) that compared outcome between a combined HITs and standard care intervention group and standard care alone control group.

## Results

### Search Results

**Fig. 1:**
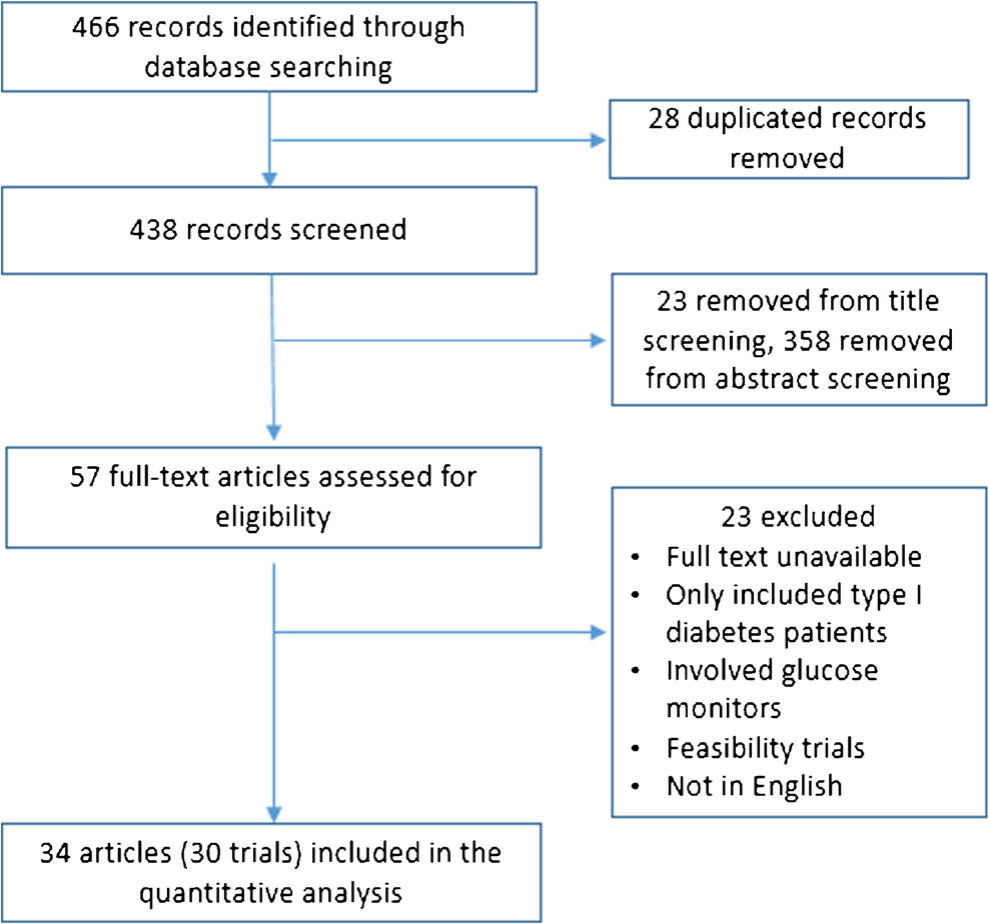
Article screening process

Figure 1 outlines the results of the search and study selection process, which identified 466 articles. A total of 381 articles were excluded with 23 based on title and 358 based on abstract. The full text of the remaining 57 were assessed with 34 articles (Quinn 2011 and Quinn 2014 are from the same trial but are analyses for different age groups; Kim HS 2007a, Kim HS 2007b, Kim HS 2008, and Kim SI & Kim HS 2008 are from the same trial but are analyses for different follow-up periods) meeting all inclusion/exclusion criteria. All 34 studies were eligible for inclusion in the meta-analysis (Fig. 1).

### Characteristics of Included Studies

Study characteristics are presented in Supplemental Table 1. The included studies were conducted in several different countries and regions: 9 in the USA [47–55], 9 in Europe [5, 32, 56–62], 8 in South Korea [63–70], 3 in China [71–73], 2 in Iran [74, 75], 1 in Canada [76], 1 in Bahrain [77], and 1 in Japan [78], The total participants were 3983, of which 2105 were randomized to intervention groups and 1878 to control groups. The majority of studies focused on type 2 diabetes (29 out of 34, 85%) [5, 47, 49–55, 57, 59–64, 66–70, 73–80]; 5 (15%) included both type 1 and type 2 diabetes [32, 48, 58, 71, 72]. The mean age for participants ranged from 30 to 60 years old. Most studies had even gender distribution; median male participation rate was 50% ranging from 22 to 96%. Eight studies (24%), 6 from the USA [47–49, 53–55], 2 from the UK [32, 79], included information on race. Eighteen out of 34 studies (53%) utilized mobile phone-based applications as intervention tools [5, 32, 48, 49, 52–55, 58, 59, 63, 64, 66–69, 78, 80]. Of these, 11 were hybrid-interventions that primarily used mobile phones to deliver treatments or services but also incorporated other applications, such as web-based applications in their programs [52–54, 58, 63, 64, 66–68, 78, 80]. Six studies (18%) used web-based applications as major intervention components [51, 60, 62, 71, 73, 76]. Seven studies (21%) used SMS/Text [47, 50, 63, 70, 74, 75, 77]. Five studies (15%) were categorized into Others, which included telephone calls or other telemonitoring devices [55, 57, 61, 72, 79]. Regarding control groups’ treatment, participants in the majority of studies (*n* = 29, 85%) received standard care and/or consultation from health care professionals [5, 32, 48, 49, 51–54, 57, 58, 60–62, 64, 66, 67, 69, 70, 72, 73, 77, 79, 80] or were engaged in diabetes self-management and/or education [50, 55, 63, 68, 76, 78]. One study compared effects of text/SMS plus pedometer vs. pedometer alone in monitoring and promoting physical activities between intervention and control groups [75]. One study compared the effects of text/SMS vs. telephone in tracking type 2 diabetes management between the two groups [47]. Three studies (9%) had unclear information on treatment for control groups [59, 71, 74]. With regard to intervention delivery personals, 10 studies (29%) had combination of medical care providers to deliver intervention [5, 49, 53–55, 57, 62, 71, 73, 78]. Eight studies (24%) exclusively used nurses as intervention delivery personnel [32, 48, 50, 52, 64, 66, 79, 80]. Eight studies (24%) used a combination of personnel, but not all being medical professional [47, 51, 59–61, 68, 76, 77]. Seven studies (21%) had no clear information on this matter [58, 63, 69, 70, 72, 74, 75]. The majority of studies under review (*n* = 23, 68%) have incorporated education components in their interventions including self-care and monitoring, life style modifications and/or medication administration and adjustment [32, 47–49, 51, 55, 57, 59, 60, 62–64, 66–71, 73–76, 80]. Ten studies (30%) incorporated interactive approaches, in which patients were not only receiving one-way messaging but also engaged in two-way communication with health professionals [47, 49–52, 60, 61, 75, 77, 79]. Seventeen out of 34 studies (50%) provided blood glucose monitors [5, 32, 48, 49, 51, 53, 54, 57, 60, 61, 68, 71, 73, 76–79]. Intervention periods in reviewed studies ranged from 2 months to a year.

### Risk of Bias

The risk of bias assessment of the studies is shown in Supplemental Fig. 1. Twenty-eight (82%) of the 34 studies reported and described an appropriate method of randomization, but only 11 (32%) reported an adequate allocation concealment process. Only 7 (21%) of all studies performed blinding for participants and personnel. For a majority of studies (32, 94%), assessors either were blinded or the outcome measurement is not likely to be influenced by lack of blinding. Twenty-six (76%) of the 34 studies addressed reasons for incomplete data. All studies included all expected outcomes, including those that were pre-specified. We did not find additional sources of bias across all studies.

A funnel plot (Supplemental Fig. 2) displays a mild asymmetry, suggesting potential for publication bias. However, the result of the fail-safe *N* test indicates that an additional 2426 studies would have to be added before the loss of statistical significance would occur. This suggests that publication bias may not be a serious issue in our analysis. Moreover, the trim-and-fill method [81] shows an imputed effect size of − 0.585 (95% CI − 0.723, − 0.447), which is the same as the original effect, indicating that minor publication bias, if there is any, is not sufficient to fundamentally alter our results.

### Quantitative Results

#### Overall Synthesis of Individual Studies

**Fig. 2:**
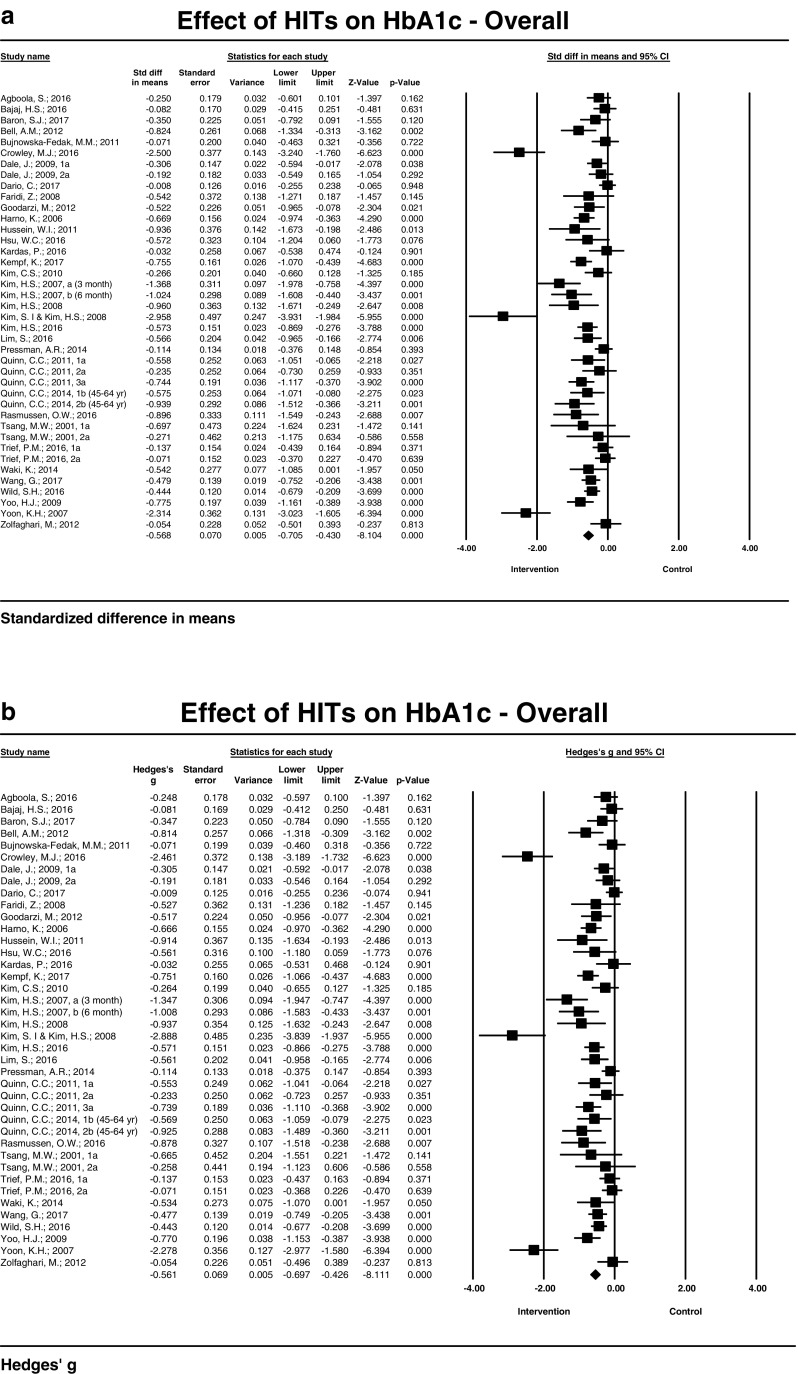
**a**. Effect of HITs on HbA1c (%): Standardized difference in mean HbA1c between intervention and control groups from baseline. **b**. Effect of HITs on HbA1c (%): bias adjusted (Hedges’g)

HIT-based strategies for patient engagement or clinical decision support included mobile, computer-based, e-mail, and internet approaches. Across 34 studies and 40 point estimates, mean basal HbA1c ranged from 6.4 to 9.9% (eAG ranges from 7.6 to 13.2 mmol/L) and 6.5 to 9.9% (eAG ranges from 7.8 to 13.2 mmol/L) in intervention and control groups, respectively. We found mean HbA1C reductions due to HITs across all studies. Reductions in HbA1C levels were statistically significant (*P* ≤ 0.05) in 25 out of 40 estimates. The mean reduction in HbA1c after intervention between intervention group and control group was − 0.65 (result not shown in table or figure). The standardized mean reduction in HbA1C resulting from HITs was − 0.57 (95% CI − 0.71, − 0.43) with values ranging from − 2.10 to − 0.01 (*I*^2^ = 76.50, Q = 165.94, *P* ≤ 0.005) (Fig. 2a). We found a random-effect adjusted effect size (Hedges’g) of − 0.56 (− 0.70, − 0.43) across all studies and estimates (Fig. 2b).

#### Overall Synthesis of Individual Studies Comparing HITs Combined with Standard Care Intervention Group Versus Standard Care Control Group

**Fig. 3:**
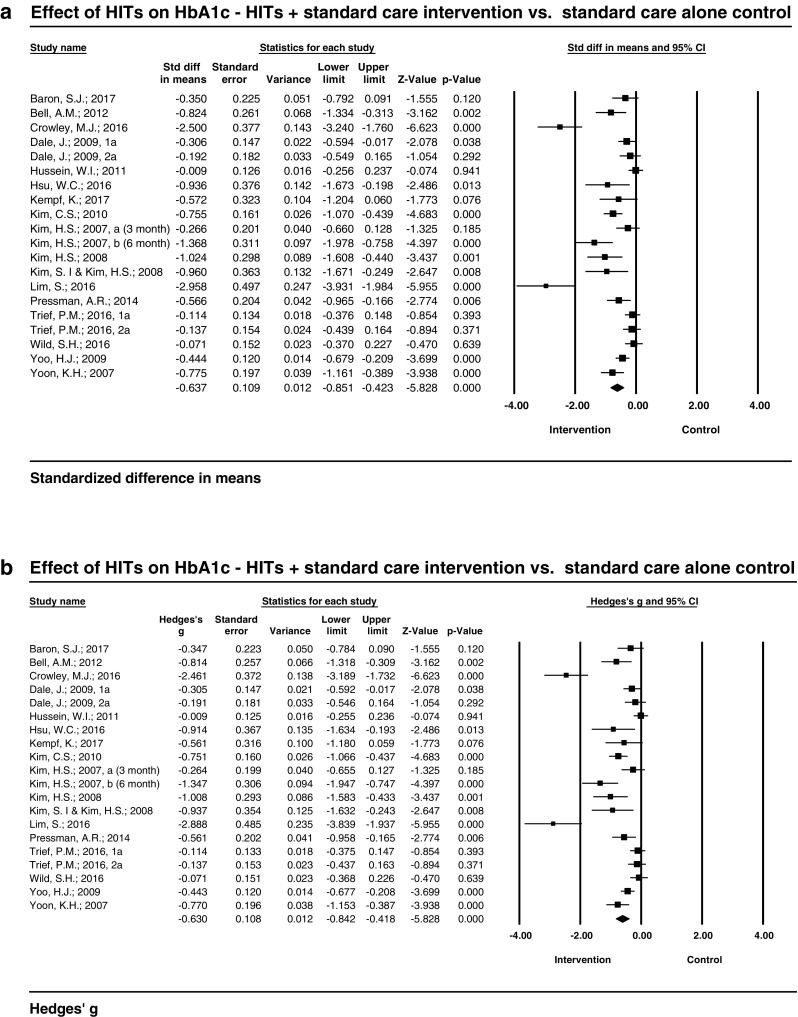
**a**. Effect of HITs on HbA1c (%): Standardized difference in mean HbA1c between HITs + standard care intervention and standard care control groups. **b**. Effect of HITs on HbA1c (%): comparison between HITs + standard care intervention and standard care control groups—bias adjusted (Hedges’g)

In this separate analysis, the standardized mean reduction in HbA1C resulting from HITs was − 0.64 (95% CI − 0.85, − 0.42) with values ranging from − 2.96 to − 0.01 (*I*^2^ = 83.05, *Q* = 112.08, *P* ≤ 0.005) (Fig. 3a.). The overall effect size (Hedges’ g) of HITs on HbA1c was − 0.63 (− 0.84, − 0.42), which can be thought as the contribution of HIT tools in addition to standard care (Fig. 3b).

#### HIT Subgroup Analysis

Table 1 presents results from HIT intervention subgroup analysis. All four categories of intervention strategies showed significant effect in reducing HbA1c. From largest to smallest effects, we found pooled standardized decreases in HbA1c for mobile-base applications, − 0.67 (− 0.90, − 0.45); SMS/Text interventions, − 0.64 (− 1.09, − 0.19); web-based applications, − 0.48 (− 0.65, − 0.30); and Others, − 0.19 (− 0.34, − 0.04). The first, second, and third largest effect sizes adjusted by random-effects model (Hedges’ g g) were − 0.66 (− 0.88, − 0.45), − 0.63 (− 1.07, − 0.19), and − 0.48 (− 0.65, − 0.30), for interventions based on mobile applications, SMS/Text, and web-based applications, respectively (data not presented in tables or figures).

**Table 1:** Intervention subgroup analysis

Intervention subgroups	Number of estimators	Standardized difference in mean HbA1c (95% CI)	*P* value	*Q*	*I*^2^%
Mobile phone-based	21	− 0.67 (− 0.90, − 0.45)	< 0.001	105.11	80.97
Web-based	6	− 0.48 (− 0.65, − 0.30)	< 0.001	8.95	44.15
SMS/text	7	− 0.64 (− 1.09, − 0.19)	0.005	33.45	82.06
Others	8	− 0.19 (− 0.34, − 0.04)	0.013	9.00	22.19

## Discussion

### Main Findings and Implications

Findings from this meta-analysis suggest that HITs lead to improvement in glycemic control, both clinically and statistically. All four intervention groups in the reviewed trials experienced a reduction in HbA1c, among which 25 out of 40 estimates (62.5%) were statistically significant. We found an overall adjusted effect size (Hedges’g) of 0.56 (− 0.70, − 0.43). This effect size is larger than what was reported in previous review studies [21•, 25–28, 37, 82], which also concluded that HITs are beneficial for glycemic control. The difference in effect size may be due to the variation in intervention types reviewed, for which we covers an array of technology-driven interventions including mobile-based, web-based, SMS/text and other approaches that aid in monitoring and communication. Other reviews only focused on a single feature of technology, such as mobile phone or telemonitoring [30, 31, 37, 82]. Or they used a general search term “informatics” [25] or information technology [22]. Also, the effect size difference may be attributed to different patient populations assessed between all reviews. In the current study, we primarily focused on a generic patient population with type 2 diabetes. In contrast, other reviews targeted more specific patient groups such as stoke survivors [37], low-income or underserved populations [27], or a mixed patient pool with both type 1 and type 2 diabetes [21•, 22, 23, 26, 31–33]. Additionally, we exclusively focused on reviewing randomized control trials, while others also covered studies with other designs including nonrandomized controlled trials, before-after trials or cross-over trials [22, 23, 25, 82]. Regarding bias due to intrinsic differences among populations of the original studies included in meta-analysis, our finding of very similar effect size measures between estimations indicates this bias was very small.

Our analysis detects a significant clinical impact of HITs in reducing dysglycemia. Evidence suggests that every 1% decrease in HbA1c over a ten-year period is associated with a risk reduction of 21% for diabetes-related death and 37% of microvascular complications [83]. This reduction results from HIT interventions may be bigger than effects of many pharmacological approaches alone. A meta-analysis examining the effect of oral antidiabetic agents (OADs) reported reductions in HbA1c levels of 0.5 to 1.25%, with thiazolidinedione and sulfonylureas showing the best reduction (1 to 1.25%) [84]. Chaudhury et al. [85] summarized the HbA1c-lowing potential for a series of therapies including biguanide, reducing HbA1c by 1.0 to 2.0%, dipeptidyl peptidase 4 (DPP-IV) inhibitor, 0.5–0.8%, GLP-1 agonists, 0.5–1.5%, and TZD, 0.5–1.4%. There was a concern that the effects on HbA1c yielded from the reviewed trials were a mixed product of both HITs and standard diabetes care including medication adherence and lifestyle modifications. This concern was addressed by the analysis of 18 studies that explicitly compared outcome between a combined HITs and standard care intervention group and standard care alone control group. This analysis yielded an effect size (Hedges’ g) of − 0.63 (− 0.84, − 0.42), which can be thought as the contribution of HIT tools in addition to the usual care. This result suggests the HITs are not only tools or one component of the interventions; they are the key to the effectiveness of these trials. It is also worth noting that most of the abovementioned pharmacotherapies were using motivated patients’ sample and they cannot generate their full effects without patients’ adherence to treatment and persistence in usage. HIT approaches have a potentially significant role in addressing challenges in adherence of a pharmacological therapy or of behavioral interventions. In this perspective, HITs may be associated with additional value to these therapeutic approaches. With its advantages of being pragmatic, highly engaging, cost-effective, and scalable [86], these technologies are promising to facilitate the interactive communication between the patients and their health care providers, provide timely reminders for medication or cues for behavioral change, enhance treatment or intervention effects, and ultimately assist patients to achieve glycemic control.

In our study, a significant reduction in HbA1c was observed with the addition of all types of HIT interventions reviewed, including the use of mobile phone-based applications, web-based application, SMS/Text, and others. The effect size, however, varied among these groups. Among all technology features under review, we found mobile phone-based and SMS/Text approaches to be particularly effective in improving glycemic profile. These findings appeared consistent with findings of two previous systematic reviews. One review focused on the effect of telemedicine on HbA1c concluded that text messaging may be especially effective mechanism for linking providers and patients with diabetes; therefore, they hold great promise to influence diabetes care positively [26]. The other review, which centered on many computer-based interventions in type 2 diabetes management, reported that the mobile phone subgroup was effective in glycemic control [87], possibly by offering convenience (and therefore adherence) and considerable intensity (e.g., allowing multiple daily contacts) of intervention. Indeed, patient-specific communication by a mobile phone or phone combined with the internet is a viable, safe, convenient, and economic option to support diabetes management [77]. Many studies have reported that patients using these services did not experience significant side-effects, and were satisfied with the intervention [67, 69, 88]. In addition, from a cost perspective, mobile phone-based teleconsultations are typically less expensive than remote blood glucose monitoring, because the later often requires the purchase of medical devices and supplies to conduct the tests [39]. Therefore, the more economic mobile-based approaches may lead to better engagement and compliance among patients. As mobile phone technology advances rapidly and becomes ever more available and affordable, it could emerge as an important tool. Future research should evaluate closely its potential to improve diabetes care in comparison with other intervention methods.

The heterogeneity observed in the study is potentially explained by the wide variation of interventions included. Among trials under review, interventions ranged from simple messages providing diabetes management suggestions for patients [48] to more comprehensive interventions permitting timely communication with and instructions from diabetes care managers via phone call, SMS and telemetry device [52, 88]. Heterogeneity may also have been affected by variations in intervention designs, the type of care or services offered to the control groups, and variations in sample composition (e.g., nationality, age, race/ethnicity).

### Limitations

One limitation of the study is that more than 80% of the trials included did not provide clear information on blinding to participants and personnel on outcome measurement. However, evidence suggests that lack of blinding is unlikely to influence an objectively assessed outcome such as HbA1c [89]. Another limitation is that the intervention effects from the HITs might be confused with the effects from the standard diabetes care and education that are given in the intervention. However, as we discussed previously, the majority of the reviewed studies included standard care to both intervention (i.e., HIT and standard care) and control (i.e. standard care alone) groups. Therefore, the effect measures for this subset of trials represent the additional effect of HITs on HbA1c. This can be partially attributed to the innovative and convenient features of treatment delivery with HIT support.

Further, our study did not include papers published in non-English language nor trial registry data, which can be another source of publication bias. Otherwise, broad inclusion/exclusion criteria were used to increase the likelihood of capturing relevant studies, including a hand search of reference lists of eligible articles, relevant systematic reviews, and narrative reviews. Moreover, while results from our risk-of-bias assessment helps to highlight the quality of the reviewed trials, unobserved confounding may also influence findings. Lastly, long-term evaluation of HITs’ effect on HbA1c warrant further research. Previous evidence showed that antidiabetic drugs achieved their maximal effect in lowering HbA1c by 3 to 6 months [84]. Most of studies in our review ranged from 2 to 12 months and they yielded a statistically significant reduction in HbA1c; however, as patients’ bodies, treatment, and circumstances change over time, whether their needs are being met along these changes and whether the effects of HITs can last are unclear [90].

## Conclusions

Our meta-analysis shows that overall, HITs have favorable impact on glycemic control of patients with type 2 diabetes. On average, mobile-based interventions produce larger effects compared to other forms of approaches under review. Our findings have critical clinical implication since HbA1c is the strongest predictors of disease progression and development of microvascular and macrovascular complications in patients with type 2 diabetes [91]. This review suggests a great incentive for further implementations of HIT-based approaches in the care of patients with diabetes. Future studies should examine the long-term effects of HITs and their cost-effectiveness, and explore factors that influence their effectiveness in glycemic control.

## Electronic supplementary material

Supplementary Figure 1 (DOCX 15 kb)

Supplementary Figure 2 (PPTX 41 kb)

Supplementary Table 1 (XLSX 19 kb)
