# Supplementary material for: Effect of Health Information Technologies on Glycemic Control Among Patients with Type 2 Diabetes
Source: Curr Diab Rep. 2018 Oct 18;18(12):130. doi: 10.1007/s11892-018-1105-2 (PMC6209028; doi:10.1007/s11892-018-1105-2)
Supplement: Supplementary file 1 — (DOCX 15 kb) [file 11892_2018_1105_MOESM1_ESM.docx]

**Figure 1. Risk of Bias Assessment**
