# Supplementary figures and images for: Effect of Health Information Technologies on Glycemic Control Among Patients with Type 2 Diabetes
Source: Curr Diab Rep. 2018 Oct 18;18(12):130. doi: 10.1007/s11892-018-1105-2 (PMC6209028; doi:10.1007/s11892-018-1105-2)

## Slide 1
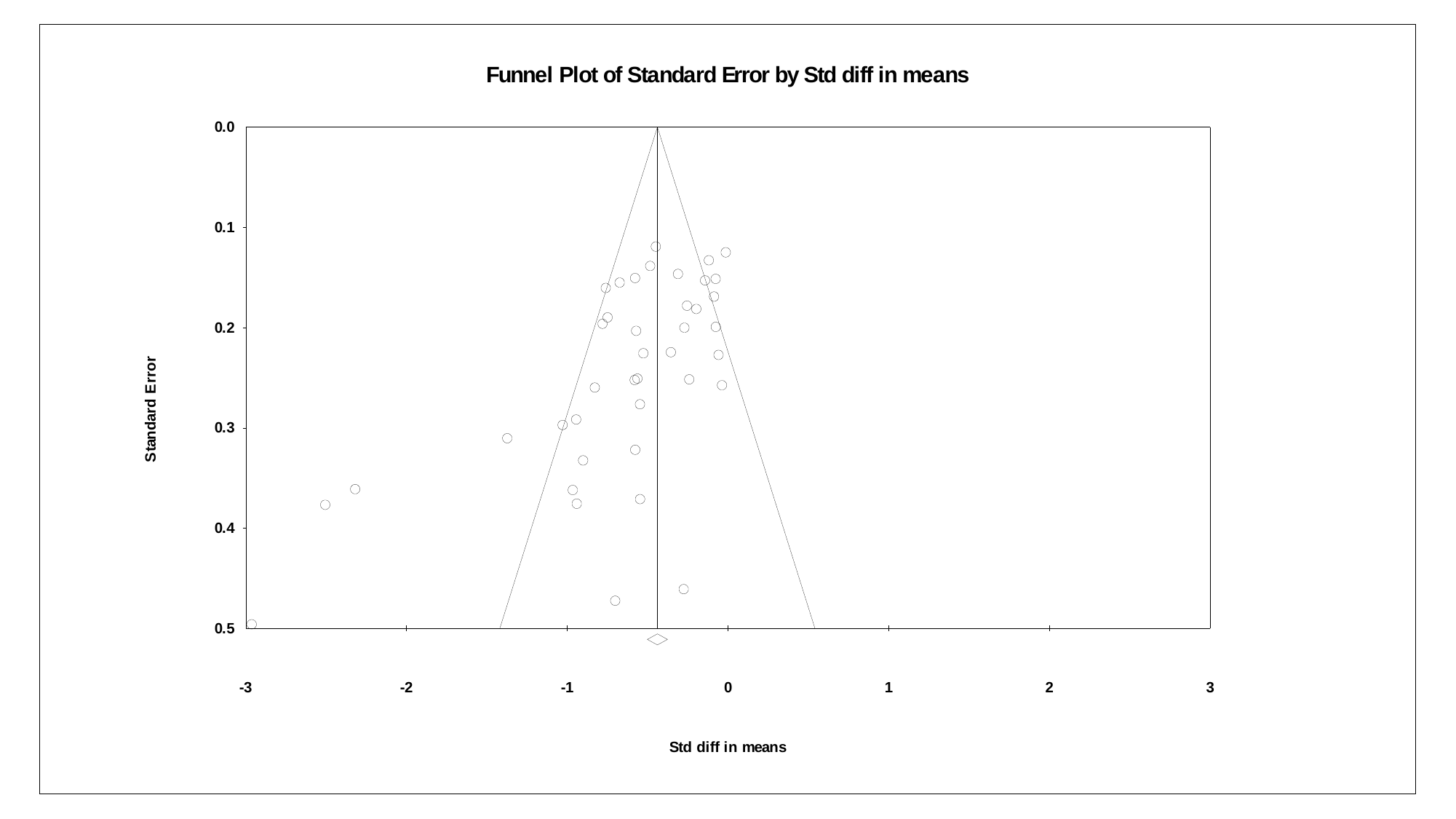

Supplement: Supplementary file 2 — (PPTX 41 kb) [file 11892_2018_1105_MOESM2_ESM.pptx]
